# Case report: Diabetic muscle infarction with diabetic ketoacidosis: A rare complication of diabetes

**DOI:** 10.3389/fendo.2022.1112703

**Published:** 2023-01-12

**Authors:** Jun Tang, Li Sun, Qi Huang, Yu-Wen Wu, Xin Li, Hao-Hua Deng, Jia-Zhong Sun, Zhe Dai, Yan-Cheng Xu

**Affiliations:** Department of Endocrinology, Zhongnan Hospital of Wuhan University, Wuhan, Hubei, China

**Keywords:** diabetic muscle infarction, diabetic ketoacidosis, pathogenesis, complication, treatment

## Abstract

**Background:**

Diabetic muscle infarction (DMI), which is also referred to as diabetic myonecrosis, is a rare and long-term complication of poorly controlled diabetes mellitus, while we found that acute diabetes decompensation, such as diabetic ketoacidosis (DKA), could also stimulate the occurrence and development of DMI.

**Case presentation:**

A 23-year-old woman with type 1 diabetes presented with a 10-day history of nausea, vomiting, pain, and swelling of her left leg. Her urine ketone test was positive. The 3-beta-hydroxybutyrate and leukocyte counts and creatine kinase levels were elevated. Magnetic resonance imaging of the left thigh revealed extensive deep tissue oedema and an increase in the T2 signal in the involved muscles. Once the diagnosis of DMI was made, she was managed with rest, celecoxib, clopidogrel and aggressive insulin therapy. Three months after treatment, the patient reported complete resolution of symptoms.

**Conclusion:**

DMI is a rare DM complication with a high recurrence rate, commonly presenting with chronic complications, while our case report shows that acute diabetes decompensation, such as DKA, can stimulate the occurrence and development of DMI. Timely diagnosis and appropriate treatment could shorten the recovery time.

## Introduction

Diabetic muscle infarction (DMI), which is also referred to as diabetic myonecrosis, is a rare and long-term complication of poorly controlled diabetes mellitus. Angervall and Stener described it in 1965 for the first time and fewer than 200 cases have been reported in the literature (1). It is typically characterized by lower-limb pain that has an acute onset. The diagnosis and treatment of DMI are often delayed as a result of its nonspecific clinical presentation (1). Diabetic ketoacidosis (DKA) is one of the most common serious acute complications of diabetes; it most often occurs in people with uncontrolled type 1 diabetes and is often accompanied by varying degrees of circulatory volume loss (2). Although most cases of DMI occurring in association with other chronic complications of diabetes have been reported (1), few cases of associated DKA have been reported thus far. We present a case of a 23-year-old woman presenting with vomiting, nausea, left leg pain and swelling who was diagnosed with DKA and DMI, and our review covers the risk factors, as well as the general prognosis and management, of the disorder. Diabetic patients with poorly controlled glucose and limb pain should be kept on high alert for DMI.

## Case presentation

A 23-year-old woman with a 9-year history of type 1 diabetes was admitted with vomiting, nausea, pain and swelling of her left leg for 10 days. She was diagnosed with DKA and treated in the local hospital for 3 days without significant improvement; then, she was transferred to our hospital for further management. The patient denied prior episodes of leg pain or trauma without medication with statins, and no peripheral vascular disease was reported. She was treated with insulin to control her glucose level. Her pain worsened with exertion and improved partially with rest. She denied fevers, chills, and weakness of the left leg.

On physical examination, vital signs were notable for temperature of 36.4°C, blood pressure of 116/93 mm Hg, heart rate of 120 bpm, height of 152 cm, weight of 50 kg, and BMI of 21.6 kg/m^2^. Other significant findings included swelling of the left leg, with mild warmth and without erythaema. On palpation, there was mild difficulty in the left leg. The pain restricted her muscle strength and motion of the left leg. The dorsalis pedis pulses were good bilaterally. She had no other musculoskeletal symptoms.

**Figure 1:**
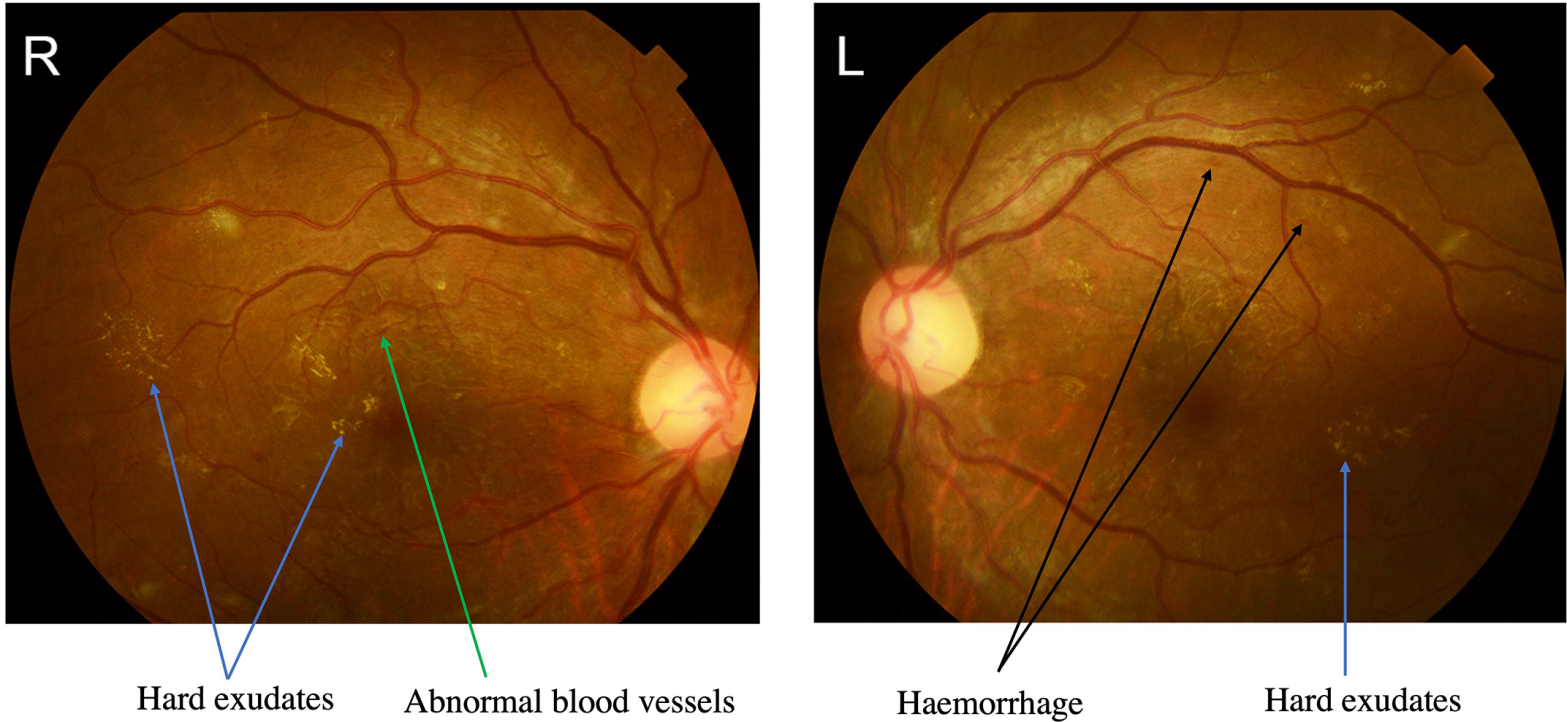
The fundus photographs showed severe proliferative diabetic retinopathy (DR). Proliferative DR showing abnormal blood vessels with green arrow, hard exudates with blue arrows and haemorrhage with black arrows.

Her haemoglobin A1c was 13.7%, random glucose was 18.1 mmol/L, and 3-beta-hydroxybutyrate was 3.67 mmol/L. Her urine acetone bodies were 3+, and her urine protein was 2+. There was mild leucocytosis, and the white blood cell (WBC) count was 9.7× 10^9/L, with 69% neutrophils. She developed mild anaemia, and her haemoglobin (Hg) level was 99 g/L. Serum electrolytes and serum creatinine were within the normal ranges. Her erythrocyte sedimentation rate (ESR) was 95 mm/h, and her highly sensitive C-reactive protein level was 442.9 mg/L; both were elevated. Her creatine kinase (CK) was 1797 U/L on admission and decreased to 1356 U/L after 3 days. She also had a mildly reduced level of serum albumin (32 g/L). Her D-dimer level was normal. Her antinuclear antibodies and anticardiolipin antibodies were normal (**Table 1**). The fundoscopic examination shows haemorrhage, hard exudates and abnormal blood vessels and proliferative diabetic retinopathy (DR) was diagnosed (**Figure 1**).

**Table 1:** Laboratory findings.

Parameters	Value	Normal range
Complete blood count		
White blood cells (× 10^9/L)	9.7	3.5-9.5
RBC Red blood cells (× 10^12/L)	5.2	3.8-5.1
Haemoglobin (g/L)	99.0	115-150
Platelets (× 10^9/L)	297.0	125-350
Neutrophils (%)	69.0	40-75
Lymphocytes (%)	16.6	20-50
Monocytes (%)	12.9	3-10
Eosinophils (%)	1.1	0.4-8
Basophile (%)	0.4	0-1
Chemistry panel		
Glucose (mmol/L)	18.1	3.9-6.1
Haemoglobin A1c (%)	13.7	<6.5
3-beta -hydroxybutyrate (mmol/L)	3.67	0-0.28
Albumin (g/L)	32.0	40-55
Alkaline phosphatase (U/L)	128	30-128
Blood urea nitrogen (mmol/L)	3.6	2.8-7.6
Creatinine (mmol/L)	54.7	49-90
Uric acid (mmol/L)	325.0	155-357
Total cholesterol (mmol/L)	3.68	<5.18
Triacylglycerol (mmol/L)	1.45	<1.7
High sensitivity C-reactive protein (mg/L)	442.9	0-300
Creatine kinase (U/L)	1797	<145
Creatine kinase-MB (U/L)	70	0-25
Hypersensitivity troponin I (pg/mL)	1.7	0-26.2
Myoglobin (ng/mL)	248.5	<140.1
Lactate dehydrogenase (U/L)	306	110-245
Lactate (mmol/L)	4.92	0.5-2.5
Procalcitonin (ng/mL)	<0.05	<0.05
Erythrocyte sedimentation rate (mm/h)	95	0-20
Blood coagulation function		
Prothrombin time(S)	11.4	9.4-12.5
International normalized ratio	1.04	0.85-1.15
Thrombin time (S)	14.3	10.3-16.6
Fibrinogen-C (mg/dL)	757	238-498
D-dimer (ng/mL)	498	9.4-500
Anti-proliferating cell nuclear antigen	–	–
Antinuclear antibodies	–	–
Rheumatoid factors (kU/L)	<10.1	0.0-15.9
Anticardiolipin antibody-IgM (MPL/mL)	0.419	<12
Anticardiolipin antibody-IgG (GPL/mL)	3.955	<12
Anticardiolipin antibody-IgA (APL/mL)	1.544	<12
Insulin autoantibody	22.8	<20
Glutamic acid decarboxylase antibody	4.948	<30
Urine routines		
Glucose	++++	–
Ketones	+++	–
Protein	++	–
Leukocytes	+++	–

**Figure 2:**
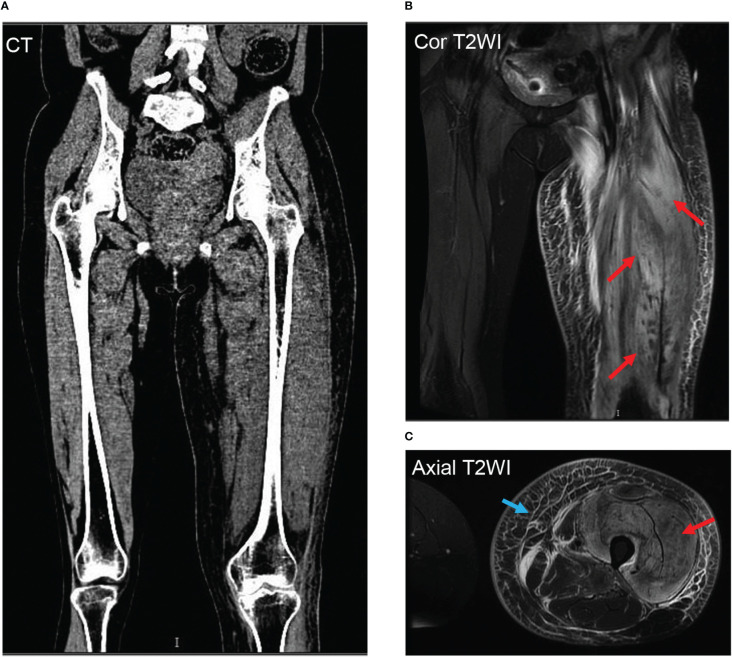
Representative thigh muscle images of conventional coronal computerized tomography (CT) image and synthetic weighted images (T2WI) with fat suppression (FS) show severe soft tissue swelling in the left thigh. The CT images **(A)** confirmed muscle swelling but no bone involvement of the femur. The corresponding areas of significant edema muscle with slightly increased signal intensity are also indicated on a coronal **(B)** and axial T2WI **(C)** with red arrows. The swelling muscle shows a slightly increased signal intensity on the T2WI map; A marked T2 hyperintensity occurred in the subcutaneous fascia of the left thigh on axial T2WI -FS with a blue arrow **(C)**.

Bilateral venous Doppler ultrasound revealed no abscess or deep vein thrombosis (DVT). Conventional coronal computerized tomography (CT) image and synthetic weighted images (T2WI) with fat suppression (FS) show severe soft tissue swelling in the left thigh. The CT images (**Figure 2A**) confirmed muscle swelling but no bone involvement of the femur. The corresponding areas of significant edema muscle with slightly increased signal intensity are also indicated on a coronal (**Figure 2B**) and axial T2WI (**Figure 2C**). The swelling muscle shows a slightly increased signal intensity on the T2WI map; A marked T2 hyperintensity occurred in the subcutaneous fascia of the left thigh on axial T2WI -FS (**Figure 2C**).

Intravascular volume correction and intensive insulin therapy were administered to treat the DKA of the patient. After the diagnosis of DMI was made, the treatment for the patient was rest, celecoxib, clopidogrel and insulin, and she improved after 10 days of treatment, with mild left leg swelling and pain. The patient was discharged with instructions for rest, celecoxib, clopidogrel and insulin. After taking celecoxib for one month and clopidogrel for three months, she recovered completely.

## Discussion

Diabetic muscle infarction, which commonly presents with swelling and pain in the lower limb muscles, is a rare complication associated with poorly controlled diabetes mellitus (1). Neither the epidemiology nor pathophysiology of DMI is fully understood. A systematic review showed that the mean age at presentation for all DMI cases was 44.6 years old (1), demonstrating a female predominance in patients with DMI. For those with type 1 diabetes (T1DM), the mean age at presentation was 35.9 years old, while for those with type 2 diabetes (T2DM), the mean age at presentation was 52.2 years (1) old. For T1DM, the mean DM duration at the time of DMI diagnosis was 18.9 years, and for T2DM, it was 11.0 years (1). DMI most commonly affects the thigh without fever or trauma history. Previous reviews have identified thigh pain/swelling as the initial presentation in 83.7% and 80% (1) of cases. It was also common to experience calf pain/swelling as a presentation of DMI, and the thigh was the most commonly affected site (75–83.7%) (1, 3), while the calf was the second most commonly affected site (15–19.28%) (3). The patient was 23 years old and had T1DM for 9 years. According to Horton et al., 46.6% of patients had concurrent retinopathy, nephropathy, and neuropathy, and 65.8% had at least two complications, which suggests that DMI is often seen in patients with long-standing diabetes, indicating that DMI is frequently associated with severe microvascular complications (1). Our case showed positive urine protein and proliferative diabetic retinopathy, indicating that the patient suffered from chronic diabetic complications with poor glucose control.

The pathophysiology of DMI remains unclear. DMI can be caused by atherosclerosis, diabetic microangiopathy, vasculitis with thrombosis, or ischaemia-reperfusion injury (4). These conditions are associated with long-term poor glucose control with chronic complications. How acute complications such as DKA induce DMI has rarely been reported, and the pathophysiology remains unknown. A case of acute DMI subsequent to diabetic ketoacidosis was reported (5), which mentioned that the dehydration caused by DKA might have induced DMI, and no chronic diabetic complications were present in this patient. In addition to dehydration, some mechanisms might explain the occurrence of DMI in acute complications. Several studies have focused on myocardial infarction in patients with decompensated diabetes, such as those with DKA (6). As a result of hyperviscosity and increased coagulation, DKA precipitates a prothrombotic environment (7). In DMI, hypercoagulability and damage to vascular endothelial cells have been identified as changes in the coagulation-fibrinolysis system (8). Ketoacidosis can also cause coronary vasospasm due to endothelial dysfunction (9). In fact, even compensated diabetic patients with chronic hyperglycaemia have subclinical myocardial injury (10). Therefore, this type of injury might be detected in the other muscles of the body and is a reason why DMI always occurs in those patients with poor glucose control. In the setting of DKA/HHS, increased counterregulatory hormone levels result in an increase in myocardial oxygen, leading to a supply-demand mismatch, resulting in myocardial necrosis (11). The release of free fatty acids was also observed in acute diabetic decompensation (11, 12). By increasing the concentration of free fatty acids in the blood, fatty acids are incorporated into the lipid structure of the myocyte membrane, causing micelles to form, in turn causing the membrane to destabilize and rupture (13). This phenomenon of cellular toxicity may induce muscle infarction (14). These factors could all be involved in the occurrence and development of DMI in these patient, and acute factors could be a new pathogenesis for DMI (**Table 2**). It is important to exclude antiphospholipid syndrome, especially in patients with T1DM, due to its long-term implications (15), but the antiphospholipid test was negative in our patient.

**Table 2:** Summary of possible pathogenesis of DMI.

Acute factors	Chronic factors
Hypercoagulability	Atherosclerosis
Endothelial dysfunction	Diabetic microangiopathy
Cellular toxicity	Vasculitis with thrombosis
	Ischaemia-reperfusion injury

In diabetic patients who present with lower extremity swelling and pain, DMI should be suspected. As a result of the nonspecific clinical findings of DMI, which are often mimicked by other conditions, such as DVT, pyomyositis, muscle abscesses, muscle neoplasms, statin-induced myopathy, peripheral vascular disease, osteonecrosis/necrotizing fasciitis, and haematomas, many cases could go undiagnosed or treated inappropriately (16). As a differential diagnosis for leg pain, rhabdomyolysis should be considered, which involves rapid breakdown of the skeletal muscles, allowing toxic cellular contents to leak into the circulation (17). Trauma, lipid lowering drugs, and infections are the most reported causes of rhabdomyolysis in adults, and our patient did not present these kinds of causes. Muscle pain, weakness, and dark tea-colored urine are classic clinical symptoms of rhabdomyolysis (17). In our case, the patient did not present left leg muscle weakness and the urine colour was normal. Acute kidney injury (AKI) is the most common systemic complication of rhabdomyolysis (17), and creatinine of our patient was normal. The pathogenesis of rhabdomyolysis is different from DMI. Rhabdomyolysis follows a common pathophysiological pathway, regardless of its cause. A muscle cell is affected either by direct damage to its membrane or by exhaustion of its energy reserves. Proteases and apoptosis pathways are activated by free ionized calcium entering the intracellular space. Ultimately, mitochondrial dysfunction results in cell death due to the production of reactive oxygen species (17).

No medication history of statins or peripheral vascular disease was reported by this patient. A lack of discriminating laboratory findings further compounds this diagnostic uncertainty. Laboratory investigations for DMI are relatively nonspecific (18). The white cell count (WCC) was within normal limits in 56.6% of cases, elevated in 42.5%, and decreased in 0.9%. In 67 of 126 cases, creatine kinase (CK) values were reported, and 68.4% were within normal limits. There is some evidence that erythrocyte sedimentation rate (ESR) and C reactive protein (CRP) levels could aid in the diagnosis of DMI. In 60 of 126 cases, ESR values were elevated by 83.3%. Among the 30 reported cases, 27 (90%) had elevated CRP. An autoimmune workup was performed in only a few patients (1).

Diabetic limb pain can be associated with elevated white blood cell count, creatinine kinase, and inflammatory markers. The affected limb usually has an abnormal linear echotexture on ultrasonography (19). This review found that eighty-two of 83 (98.8%) cases had negative DVT ultrasonography findings (1). There are fewer specific CT findings in DMI than on MRI, but muscle enlargement with diminished attenuation, thickening of adjacent fascial planes and skin overlying the infarction, and increased subcutaneous attenuation could be observed (3). MRI has been shown to be an effective diagnostic tool in recent studies. In fat-suppressed T-2 weighted images, abnormal MRI findings have shown a sensitivity close to 100%. Diagnosis can be made with MRI due to its sensitivity and specificity (3). T2-weighted images usually show hyperintensity, whereas T1-weighted images usually show isointense to hypointense signals from the affected muscle, with associated subcutaneous, perifascial, and/or perimuscular oedema (3).

It is the gold standard of diagnosis to perform a muscle biopsy, but this procedure might not be necessary if classic history and MRI findings are present. Due to the increased time to symptom improvement and procedure-associated complications, muscle biopsy is not recommended for definitive diagnosis at this time (20). The pathology specimens for muscle biopsy usually show necrosis and oedema (1).

Intensive glycaemic control, rest, and NSAID treatment are the major recommendations for treatment. Shortening recovery time and improving pain might be possible with NSAIDs. The combination of rest plus NSAIDs resulted in a short recovery time. The antiplatelet therapy aspirin is commonly prescribed, but evidence regarding its efficacy is equivocal (1), and our patient was treated with clopidogrel. Physical therapy and surgery are not recommended (1) Physical therapy could prolong recovery time unless the lesion is fully healed. DMI patients with surgery had a mean time to symptom resolution of >80 days compared to patients with bed rest of approximately 40 days, and patients who underwent surgery had a higher recurrence rate (1). The risk of complications and recurrence should be decreased with adequate glycaemic control, rest, and NSAID use (1).

In summary, DMI is a rare DM complication with a high recurrence rate, commonly presenting with chronic complications, while our case report shows that acute diabetes decompensation, such as DKA, can stimulate the occurrence and development of DMI. Timely diagnosis and appropriate treatment could shorten the recovery time.

## Data availability statement

The original contributions presented in the study are included in the article/supplementary material. Further inquiries can be directed to the corresponding authors.

## Ethics statement

The studies involving human participants were reviewed and approved by Ethics Committee of Zhongnan Hospital of Wuhan University. The patients/participants provided their written informed consent to participate in this study.

## Author contributions

JT, ZD and Y-CX: conception and design of the work. QH, Y-WW and XL: data collection. LS, H-HD and J-ZS: Image analysis and interpretation. JT, LS manuscript writing. All authors were involved in revising manuscript critically. All authors contributed to the article and approved the submitted version.
